# Superinfection by PHYVV Alters the Recovery Process in PepGMV-Infected Pepper Plants

**DOI:** 10.3390/v12030286

**Published:** 2020-03-05

**Authors:** Myriam G. Rodríguez-Gandarilla, Edgar A. Rodríguez-Negrete, Rafael F. Rivera-Bustamante

**Affiliations:** 1 Unidad Irapuato, Departamento de Ingeniería Genética, Centro de Investigación y de Estudios Avanzados (Cinvestav) del IPN, Km. 9.6 Libramiento Norte, Irapuato, Guanajuato 36821, Mexico; myriam.rodriguez@cinvestav.mx; 2 Departamento de Biotecnología Agrícola, CIIDIR-Unidad Sinaloa, Instituto Politécnico Nacional, Guasave, Consejo Nacional de Ciencia y Tecnología (Conacyt), Sinaloa 81101, Mexico; earodriguezne@conacyt.mx

**Keywords:** geminivirus, host recovery, silencing suppressors, PTGS, TGS

## Abstract

Geminiviruses are important plant pathogens that affect crops around the world. In some geminivirus–host interactions, infected plants show recovery, a phenomenon characterized by symptom disappearance in newly emerging leaves. In pepper–*Pepper golden mosaic virus* (PepGMV) interaction, the host recovery process involves a silencing mechanism that includes both post-transcriptional (PTGS) and transcriptional (TGS) gene silencing pathways. Under field conditions, PepGMV is frequently found in mixed infections with *Pepper huasteco yellow vein virus* (PHYVV), another bipartite begomovirus. Mixed infected plants generally show a synergetic phenomenon and do not present recovery. Little is known about the molecular mechanism of this interaction. In the present study, we explored the effect of superinfection by PHYVV on a PepGMV-infected pepper plant showing recovery. Superinfection with PHYVV led to (a) the appearance of severe symptoms, (b) an increase of the levels of PepGMV DNA accumulation, (c) a decrease of the relative methylation levels of PepGMV DNA, and (d) an increase of chromatin activation marks present in viral minichromosomes. Finally, using heterologous expression and silencing suppression reporter systems, we found that PHYVV REn presents TGS silencing suppressor activity, whereas similar experiments suggest that Rep might be involved in suppressing PTGS.

## 1. Introduction

Geminiviruses are important plant pathogens that affect crops around the world. Their genomes are composed of circular, single-stranded DNA molecules packed into an icosahedral twinned particle [1]. *Geminiviridae* family is divided into nine genera (*Begomovirus*, *Mastrevirus*, *Curtovirus*, *Becurtovirus*, *Eragrovirus*, *Topocuvirus*, *Turncurtovirus, Capulavirus*, and *Grablovirus),* based on their genome organization, host range, and insect vector [2]. The begomoviruses comprise the most diverse genus with around 400 species. They infect dicots, are transmitted by whiteflies, and include monopartite and bipartite species [3,4]. Bipartite begomoviruses encode six open reading frames (ORFs) distributed into two molecules called components A and B. Component A contains the capsid protein gene CP in the virion sense strand, whereas the complementary sense strand encodes the replication-associated protein Rep (an essential protein for viral replication), TrAP (a multifunctional protein involved in the activation of late viral genes and suppressor of host gene silencing), and REn (a protein that enhances viral DNA accumulation during the replication). The component B encodes two movement proteins: an intracellular movement protein NSP, and a long-distance movement protein MP [5].

*Pepper golden mosaic virus* (PepGMV) is a whitefly-transmitted, bipartite begomovirus that infects solanaceous crops such as pepper and tomato in Mexico and Central America. After an initial stage with strong symptoms, pepper plants infected with PepGMV show a reduction of symptoms on the new leaves. This phenomenon has been called host recovery [6]. PepGMV is frequently found in mixed infections with another bipartite begomovirus, *Pepper huasteco yellow vein virus* (PHYVV). This mixture has been detected in both cultivated and wild peppers [7,8,9]. Interestingly, co-infection assays revealed that this mixed infection results in a synergic interaction, increasing DNA concentration of both viruses, without any noticeable effect on the localization of either virus on infected plants. Additionally, pepper plants with PepGMV/PHYVV mixed infection are unable to show the recovery phenotype [8].

RNA silencing is an important gene regulation mechanism conserved in eukaryotic organisms; in plants, it plays a key role in antiviral defense. Viral dsRNA is recognized by DICER-like proteins and processed into 21 to 24-nt viral-derived small RNAs, which form different complexes with Argonaute (AGO) proteins guided to their corresponding targets in a sequence-specific manner. The silencing process can act at two levels, a post-transcriptional gene silencing level (PTGS) that directs viral and cellular mRNA degradation, and a transcriptional gene silencing level (TGS) by RNA-directed DNA methylation (RdDM) and viral chromatin modifications, to restrict viral replication, transcription, and proliferation [10,11]. Geminiviruses have been reported as targets of both gene silencing pathways, PTGS and TGS [12,13,14]

Geminiviruses replicate in the nucleus of an infected cell by a rolling circle replication mechanism that involves a double-stranded replicative form (RF) intermediary. This intermediary constitutes the template for viral replication and transcription. Viral RNAs are targeted for degradation by a PTGS mechanism [15]. On the other hand, viral RFs are associated with cellular histones to form minichromosomes [16,17], and similar to plant chromatin, viral minichromosomes suffer various DNA and histone modifications guided by TGS mechanism [18]. These modifications could affect viral gene expression. For example, Histone 3 lysine 4 trimethylation (H3K4me3) is associated with transcriptionally active euchromatin, while H3K9me2 and H3K27me3 are repressive marks [19].

As a counter defense, many plant viruses encode suppressors of RNA silencing (VSR), which interfere with silencing pathways using various molecular mechanisms [20,21,22]. Identified geminivirus-encoded VSRs included TrAP, L2, C4, V2, Rep, and C5. These viral proteins can disrupt both PTGS and TGS defensive pathways, interfering at different points: reduction in siRNA synthesis, sequestration of siRNA molecules, interaction with AGO proteins, inhibition of enzymes of the methyl cycle, reduction of the expression of DNA methyltransferases, and interaction with histone methyltransferase KYP [13,23,24,25,26,27,28,29,30,31].

There is strong evidence that viral DNA methylation by RdDM is an important mechanism to regulate viral replication. The in vitro DNA methylation of TGMV reduces viral replication in tobacco protoplasts [32]. Low levels of viral DNA methylation are associated with symptomatic infection, whereas the host recovery infection phenotypes are related to hypermethylated viral genome, association to repressive histone marks and minichromosomes condensation [14,18,33]. *Arabidopsis* plants with mutations in components of RdDM pathway are unable to recover after infection with *Beet curly top virus* lacking a suppressor protein that inhibits methylation (BCTV L2^-^), and viral DNA shows hypomethylation [12,34,35,36]. In natural host–virus interaction, a recovery process also associated with gene silencing was observed in *Cucurbit leaf crumple virus*-infected watermelon [37]. Similarly, recovery from PepGMV infection was related to hypermethylation of viral genome and minichromosomes compaction in a natural pepper–PepGMV interaction [14,18].

Previous work has focused into the characterization of the PepGMV–pepper host recovery process, however, host recovery is a dynamic phenomenon that can be disrupted by synergic interaction with PHYVV. This study explores how PHYVV superinfection can reverse host recovery in PepGMV-infected pepper plants and the involvement of PHYVV-encoded silencing suppressor activity in this process.

## 2. Materials and Methods

### 2.1. Plant Material and Growth Conditions

Pepper (*Capsicum annuum* var Sonora Anaheim) plants were grown at 26–28 °C. *Nicotiana benthamiana* lines 16c (transgenic line that expresses constitutively GFP (green fluorescent protein) gene under control of 35S promoter) [38,39] and 16c-TGS plants (transcriptionally silenced GFP), were obtained as previously described [27] and maintained at 22–25 °C. All plants were grown with a photoperiod cycle of 16 h light and 8 h dark.

### 2.2. Viral Clones

PepGMV and PHYVV dimeric infectious clones have been described earlier [6,40].

To produce all recombinant PVX constructs, DNA fragments including individual PHYVV genes were obtained by PCR amplification using specific primers (Table S1 and Figure S1) using the Pfx DNA pol (Invitrogen, Carlsbad, CA, USA). PCR amplicons were cloned into PVX-containing pgR107 vector [41,42] in the SmaI restriction site, using T4 DNA ligase (Thermo Scientific, Waltham, MA, USA). The correct orientation was verified by PCR and DNA sequencing. PVX::PHYVV-gene constructs were transformed into GV3101 *Agrobacterium tumefaciens* strain for agroinoculation studies.

### 2.3. Virus Inoculation

PepGMV and PHYVV infectious clones were inoculated into pepper plants by a biolistic delivery, as previously described [6]. The first and second leaves were inoculated with tungsten particles (0.7 mm, Bio-Rad, Hercules, CA, USA) coated with viral DNA (infectious dimeric clones) accelerated by releasing Helium at low pressure (100 to 120 psi).

Recombinant PVX inoculation into *N. benthamiana* plants was carried out by an *Agrobacterium*-mediated procedure as previously described [43]. Agrobacterium cells carrying PVX-derived pGR107 vector containing the specific PHYVV gene to be tested were incubated overnight at 28 °C. The cultures were pelleted and resuspended to an optical density OD600 = 1.0 in a solution containing 10 mM MgCl_2_, 10 mM MES pH 5.8, and 100 μM acetosyringone. Cells were then incubated at room temperature for at least 4 h prior to their infiltration into *N. benthamiana* plants at the 3–4 leaf stage. Infiltration was performed by gently pressing a 1 mL syringe (containing the bacterial suspension) into the abaxial surface.

PepGMV-infected pepper plants showing recovery were inoculated with recombinant PVX constructions by mechanical inoculation [44]. To obtain the sap inoculum, leaves of infected *N. benthamiana* were ground in a 0.05 M potassium phosphate buffer solution (pH 7.5). The resulting suspension inoculum was rubbed with carborundum onto the apical leaves of pepper plants showing recovery. Inoculated leaves were then rinsed with distilled water.

### 2.4. Superinfection Assays

**Figure 1:**
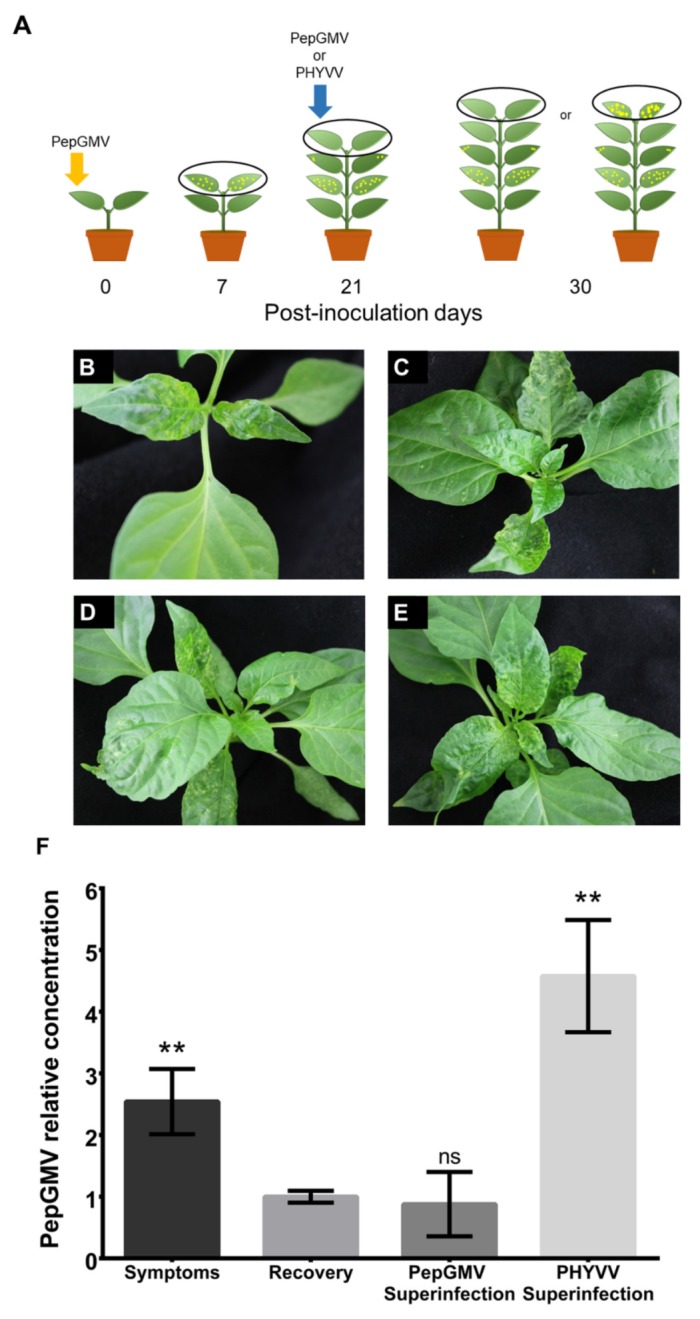
Symptom severity and *Pepper golden mosaic virus* (PepGMV) DNA accumulation. (**A**) Schematic representation of inoculation protocol. Pepper plants initially inoculated with PepGMV developed symptoms at 7 days after inoculation (dpi). Plants showing recovery (21 dpi) were superinfected with either PepGMV or *Pepper huasteco yellow vein virus* (PHYVV). Newly developed tissue was collected and analyzed at 30 dpi. Black ovals indicate leaves collected for analysis. (**B**–**E**) Symptom severity: (**B**) PepGMV symptoms, 7 dpi. (**C**) Host recovery, 21 dpi. (**D**) PepGMV superinfected plants, 30dpi. (**E**) PHYVV superinfected plants, 30dpi. (**F**) PepGMV DNA accumulation. Total DNA was isolated from leaf tissue indicated in black ovals in (A). PepGMV DNA levels were quantified by qPCR using the internal gene CaEF1a for standardization. Bars represent the relative concentration of PepGMV DNA using the value obtained in the recovered tissue as reference. Each bar represents the mean value of three independent biological replicates. Each replica is a pool of five plants. Error bars plotted refer to mean standard deviation. Asterisks indicate samples that are statistically different when compared with the recovered tissue reference (**, *p* < 0.01, Symptoms-Recovery or PHYVV-Recovery; ns, no significant, PepGMV-Recovery), as determined by Student’s *t*-test.

Pepper plants were initially inoculated with PepGMV. Twenty-one days after PepGMV inoculation, recovered pepper plants were then superinfected with either PHYVV or PepGMV. Leaf tissue was collected at 7, 21, and 30 days after inoculation (dpi) as indicated in Figure 1A. Samples were immediately frozen in liquid nitrogen and stored at −80 °C for subsequent analysis.

### 2.5. Viral DNA Accumulation in Infected Plants

Total DNA extraction was carried out according to a CTAB protocol [45]. Apical leaves from five plants were mixed and ground in liquid nitrogen. Approximately 0.3 g of leaf tissue was transferred to a microfuge tube containing 1 mL of extraction buffer (3% CTAB, 1.4 M NaCl, 20 mM EDTA, 100 mM Tris pH 8, 3% PVP), and incubated at 65 °C for 10 min. After chloroform extraction, the DNA was precipitated with 2.5 vol of ethanol, washed with 70% ethanol, and resuspended in water.

Viral DNA accumulation was determined by quantitative real-time PCR (qPCR) using the Maxima SYBR Green qPCR Master mix (Thermo Scientific) and CFX-96 Real-time system thermal cycler (Bio-Rad), following manufacturer’s instructions. The PCR protocol included an initial denaturing step at 95 °C for 10 min, followed by 35 cycles of 95 °C for 15 s and 59 °C for 60 s. The PepGMV titer determination was carried out by a relative expression protocol according to the 2^−∆∆C^_T_ method [46] using *C. annuum* Elongation factor alpha 1 (EF1-α) gene as normalizer control. The primers used are listed in Table S1.

### 2.6. Isolation of PepGMV Minichromosomes

The procedure for viral minichromosome enrichment has been previously described [18]. Briefly, 2 g of fresh tissue was ground in liquid nitrogen until a fine powder was obtained. The powder was then homogenized at 4 °C in 20 mL of 10mM Tris-HCl buffer, pH 9.0 that contained 500 mM sucrose, 80 mM KCl, 0.5 mM spermidine, 0.5 mM spermine, 0.5% Triton X-100, 10mM EDTA, and 15 mM β-mercaptoethanol supplemented with Sigmafast protease inhibitor cocktail tablet (Sigma-Aldrich, St. Louis, MO, USA). The resulting homogenate was first filtered through two layers of cheesecloth and, then, two layers of Miracloth (475855; Calbiochem). The filtrate was centrifuged for 15 min at 2000× *g* (e.g., Sorval RC-5B, ss-34 rotor). The nuclei-rich sediment was resuspended in 0.5 mL of extraction buffer (10 mM Tris-HCl pH 8 plus 0.1% Sarkosyl detergent) and immediately incubated on ice for 15 min. The suspension was then centrifugated at 2000× *g* for 15 min. The supernatant (nuclear extract) containing viral minichromosomes was recovered and saved for further analysis.

### 2.7. Methylation Density Analysis

**Figure 2:**
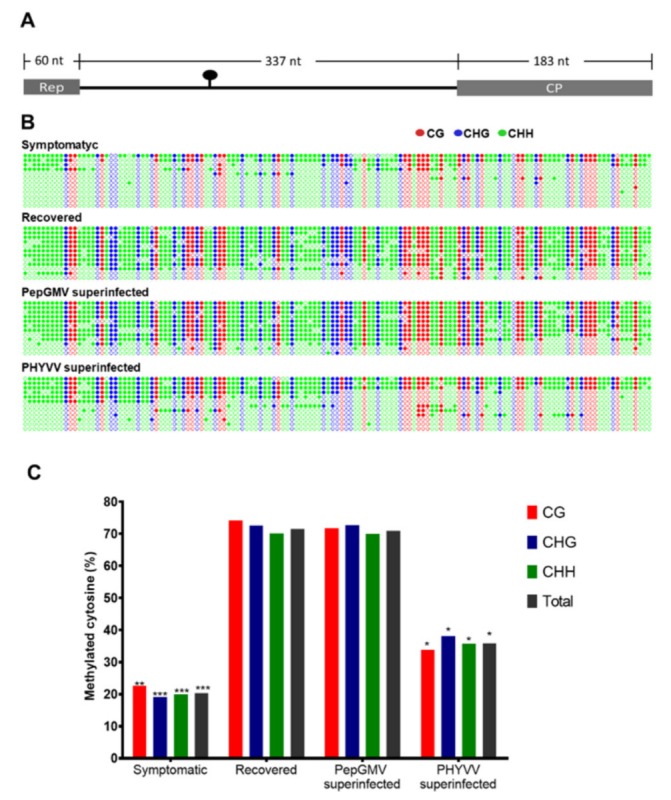
PepGMV DNA methylation level. (**A**) Schematic representation of the PepGMV 580 bp fragment analyzed. The analyzed region includes the first 60 bp of Rep coding region, the 337 bp of PepGMV A intergenic region (IR), and 183 bp of the 5′ end of CP coding region. (**B**) Methylation status of the PepGMV DNA. DNA from minichromosome extraction was treated with bisulfite, the 580 bp fragment was amplified by PCR, and PCR products were cloned and sequenced. Rows represent 12 individual clones from each treatment (organized from high to low methylation). Dot graphics represent all 138 cytosines present in the analyzed region in the following context, CG (30) in red, CHG (28) in blue, and CHH (80) in green. Filled circles indicate a methylated cytosine (mC). (**C**) Histograms show the percentage of mC residues in all sequence contexts. A Student’s *t*-test was performed using individual clones as data points. A significant difference between the samples in a pair is indicated as * *p* < 0.05 (for pair PHYVV Superinfected-Recovered), ** *p* < 0.01 (for pair Symptomatic-Recovered in CG), or *** *p* < 0.001 (in CGH, CHH, and total). Pair Recovered-PepGMV superinfected did not show significant differences.

To analyze the level of methylation of PepGMV DNA, DNA from the minichromosome-enriched supernatant was extracted by a phenol-chloroform method [47]. We mixed DNA obtained from three independent extractions of minichromosomes and 100 ng of DNA from this mix was first linearized by digestion with EcoRI restriction enzyme and then treated with bisulfite using an EZ DNA Methylation-Gold kit (Zymo Research, Irvine, CA, USA). Following manufacturer’s instructions methylation analysis was focused on a 580-bp fragment that includes the entire intergenic region (IR, 337 bp) and the 5′ends of both CP (183 bp) and Rep (60 bp) open reading frames as depicted in Figure 2A. The 580bp fragment was amplified using primers previously described [14,18]. As a control for the bisulfite conversion, 100 ng of a plasmid containing a monomeric clone of PepGMV A was mixed with an excess of plant DNA and used in a parallel bisulfite treatment reaction.

To analyze methylation level of the 35S promoter region, total DNA was extracted from plant vascular tissues using the CTAB protocol. Extracted DNA (200 ng) was digested with enzyme EcoRI, an enzyme that does not cut inside the promoter region. As in the case of viral DNA, bisulfite treatment was performed following the procedure suggested by the manufacturer (EZ DNA Methylation-Gold kit D5005, Zymo Research). After treatment, plant DNA was used as template in a PCR assay for the amplification of a 338-bp fragment of the 35S promoter using previously reported primers [48,49] with some modifications (Table S1).

PCR products obtained from the bisulfite-treated DNA were cloned using the CloneJET PCR Cloning kit (Thermo Scientific). At least 12 clones from each sample were sequenced and analyzed using Kismeth software (http://katahdin.mssm.edu/kismeth/revpage.pl) [50].

### 2.8. ChIP-qPCR Analysis

The chromatin immunoprecipitation (ChIP) protocol was based on the method described by Saleh et al. [51]. The anti-histone H3K9me2 (ab194680; Abcam, Cambridge, MA, USA) and anti-histone H3K4me3 (ab8580; Abcam) antibodies were bound to protein A dynabeads for 4 h at 4 °C.

Symptomatic, recovered, and superinfected tissues (1 g) were used for isolation of PepGMV minichromosomes (500 μL). Protein A dynabeads (Thermo Scientific) were used for pre-clearing for at least 2 h at 4 °C. After pre-clearing the minichromosomes extract was incubated with the antibody-Protein A dynabeads overnight at 4 °C. DNA was extracted using phenol-chloroform, followed by ethanol precipitation. Purified viral DNA was quantified by real-time qPCR.

### 2.9. PHYVV Gene Expression Analysis

RNA extraction was performed with TRIzol reagent (Invitrogen) according to the manufacturer’s recommendations. RNA solution was DNase treated for 15 min at room temperature using 1 U of DNaseI Amp grade (Invitrogen) per μg of total RNA. The enzyme was then inactivated by incubation at 65 °C for 10min in the presence of EDTA. cDNA was synthesized using iScript Advanced reverse transcriptase (Bio-Rad) and an incubation at 46 °C for 20 min. First-strand cDNA was used as template for PCR amplification with the gene-specific primers. As an additional control, PVX-specific primers flanking the multicloning site were used to verify the integrity of vector constructions containing PHYVV genes. EF1-α-derived amplicon was also used as PCR internal control. All primers are listed in Table S1.

### 2.10. Suppressor Silencing Analysis

To test if PHYVV complete virus or its encoded genes are TGS suppressors, *N. benthamiana* 16c-TGS plants at the 3–4 leaf stage were inoculated by biolistic with dimeric infectious clones of PHYVV, or by agroinoculation with the PVX constructs to express the PHYVV genes individually.

Agrobacterium-mediated virus inoculation was carried out as previously described [43]. Agrobacterium cultures were pelleted and resuspended to an optical density OD600 = 1.0 in a solution containing 10 mM MgCl_2_, 10 mM MES pH 5.8, and 100 μM acetosyringone, and were incubated at room temperature for at least 4h prior to infiltration.

For agroinoculation of *N. benthamiana* 16c-TGS, plants at the 3–4 leaf stage were infiltrated by gently pressing a 1 mL syringe to the abaxial surface.

PTGS assays were performed in *N. benthamiana* 16c plants (expressing constitutively GFP) by agroinfiltration-mediated transient expression, using co-infiltration of 35S-GFP construct with either pBINX expressing PHYVV Rep or REn proteins under control of 35S promoter, or pBINX empty vector as control.

GFP fluorescence was monitored (in inoculated leaves or in new emerged systemic leaves for PTGS and TGS assays, respectively) under handheld UV lamp (UVP Blak-Ray™ B-100AP), photographed with a Canon EOS RebelT3 digital camera, and tissue collected and stored at −80 °C for subsequent analysis.

### 2.11. GFP Expression Analysis

RNA extraction was performed with TRIzol reagent (Invitrogen) according to the manufacturer’s recommendations. RNA solution was DNaseI treated as mentioned before. The cDNA was synthesized with iScript Advanced reverse transcriptase (Bio-Rad) at 46 °C for 20 min.

Real-time PCR was performed by using Maxima SYBR Green qPCR Master mix on CFX-96 Real-time system thermal cycler (Bio-Rad), following the manufacturer’s instructions. The primers used are in Table S1. Relative transcript levels were obtained using the 2^−∆∆C^_T_ method [46], and NbEF1a as normalizer gene.

### 2.12. Chop-PCR Analysis

Total DNA was extracted from plant vascular tissues, and 1μg was digested with 10 U of McrBC (New England Biolabs, Ipswich, MA, USA), methylation-dependent restriction endonuclease, in a 20 μL reaction according to the manufacturer’s recommendations. The enzyme was heat-inactivated, and 1 μL of the cleaved DNA was amplified with primers for CaMV 35S promoter (Table S1). PCR products from not digested DNA were used as controls.

## 3. Results

### 3.1. PHYVV Superinfection Reverts Host Recovery in PepGMV-Infected Pepper Plants

In previous works, we have shown that pepper plants infected with PepGMV present a host recovery process three weeks after inoculation [6]. On the other hand, pepper plants simultaneously inoculated with PepGMV and PHYVV show an enhancement of the induced symptoms compared with the ones observed after individual infections (synergism). In addition, plants that show synergism do not present recovery [8]. We wondered if a superinfection with PHYVV of a recovered PepGMV-infected plant will alter the recovery process and induce synergism. To test this, we inoculated pepper plants with PepGMV and after the recovery stage was established, the plants were challenged with PHYVV as described in Figure 1A.

Seven days after the initial inoculation, plants exhibited characteristic yellow mosaic symptoms (Figure 1B). At 21 dpi (Figure 1C), the newly emerged leaves showed a reduction in the severity of symptoms. This stage, known as recovery, has been described previously [6]. Superinfection of the recovered plant with PHYVV resulted in the re-emergence and increase of symptoms (Figure 1E). In contrast, the newly developed leaves of the plants superinfected with PepGMV remained asymptomatic (Figure 1D).

To further examine if the changes in symptom severity were correlated with viral DNA concentration, we performed a qPCR to quantify PepGMV DNA in apical leaf tissue from all treatments. This data revealed that PHYVV superinfection caused a 4.5-fold increase in PepGMV DNA accumulation compared with untreated, recovered plants. On the other hand, PepGMV superinfection did not significantly alter the accumulation of viral DNA (Figure 1F).

These results indicated that the presence of PHYVV induces an increase in PepGMV viral titer that is correlated with the emergence of severe symptoms (synergism) that disrupt the host recovery stage.

### 3.2. DNA Methylation Level of PepGMV Genome is Reduced in PHYVV Superinfected Plants 

Considering that the recovery process is usually associated with viral DNA hypermethylation [14,18,34], we asked whether PHYVV superinfection could induce changes in the level of methylation of PepGMV DNA.

To answer that, we performed an enrichment of PepGMV minichromosomes from four types of tissue samples: symptomatic, recovery, and both types of superinfections, PepGMV and PHYVV, (see Figure 1A). After DNA extraction, we carried out bisulfite sequencing of PepGMV complementary strand of a 580 bp fragment encompassing 60 nt of Rep coding region, 337 nt of intergenic region (IR), and 183 nt of CP coding region as previously described [14,18].

The analyzed region (Figure 2A) contains 138 cytosines in the following contexts: 30 CG, 28 CHG, and 80 CHH (where H is any nucleotide except G). Cytosine methylation profiles representing individual PepGMV bisulfite sequencing clones obtained from all different tissue samples are shown in Figure 2B. Twelve independent clones were analyzed from each type of tissue.

It is interesting to highlight that, in a given plant sample, two types of viral genome populations can be found, one highly methylated and one almost unmethylated. The overall percentage of methylation depends on which of the two populations represent the majority in the analyzed stage (symptom, recovery, or superinfection).

Bisulfite sequencing of viral DNA obtained from symptomatic and recovered tissues confirmed that level of DNA methylation (methylated cytosines, mC) is higher in recovered tissue than the level obtained with symptomatic tissue in all sequence contexts (71% and 20%, respectively) (Figure 2C). Remarkably, similar hypermethylation (70% mC) was observed in PepGMV superinfected tissue but not in the equivalent sample from PHYVV superinfection where considerably lower methylation was observed (35% of mC). These results confirm that viral DNA hypermethylation is a hallmark of recovered tissue.

### 3.3. CHIP-qPCR Analysis

**Figure 3:**
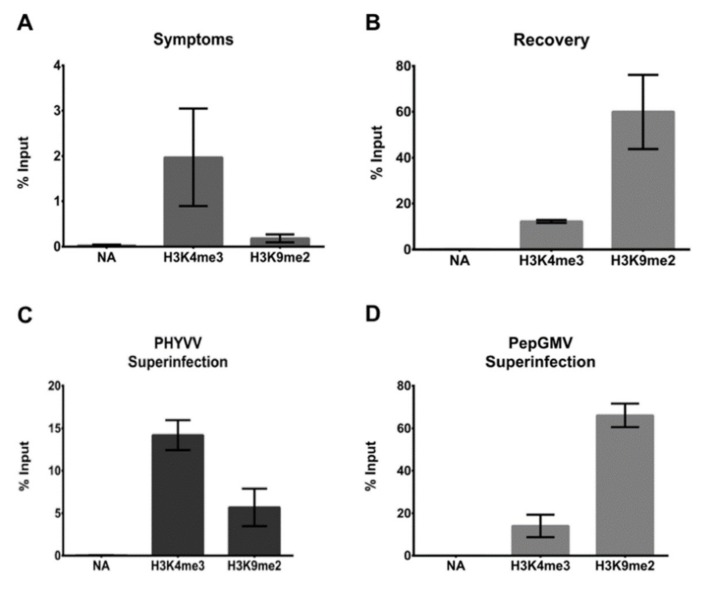
ChIP-qPCR analysis of the association of H3K4me3 (active) and H3K9me2 (repressive) histone marks with PepGMV minichromosomes. After minichromosome enrichment from symptomatic (**A**), recovered (**B**), recovered PHYVV-superinfected (**C**), or recovered PepGMV-superinfected (**D**) tissue, an immunoprecipitation assay was carried out using antibodies against H3K4me3 or H3K9me2, and pulled down viral DNA was quantified by qPCR using PepGMV IR specific primers. The data are presented as percentages of the input. Bars represent mean values of three independent biological replicates; error bars plotted refer to standard deviation of the mean. NA, no antibody

Geminivirus dsDNA RF associates with host histones to assemble into a minichromosome structure that can be affected by epigenetic regulation [16]. To verify the chromatin state of PepGMV minichromosomes after superinfection with PHYVV, we conducted chromatin immunoprecipitation (ChIP) analyses of two well characterized histone marks: H3K4me3, reported in active chromatin and H3K9me2, reported associated to a repressive state of chromatin. After viral minichromosome enrichment, viral DNA was pulled down with the respective antibodies (anti-H3K4me3 or anti-H3K9me2), and quantified by a qPCR procedure targeting PepGMV IR. The obtained results confirm that in symptomatic tissue more viral DNA is associated with H3K4me3 activation mark than to the repressive mark H3K9me2 (Figure 3A), On the other hand, in recovery tissue viral DNA is preferentially associated with the repressive mark H3K9me2 (Figure 3B). Results from the superinfection experiments showed that in the case of PHYVV superinfection, the pattern between H3K4me3 and H3K9me2 (Figure 3C) is similar to the one obtained with symptomatic tissue (Figure 3A). On the other hand, in the case of PepGMV superinfection, the pattern observed (Figure 3D) is similar to the one observed with recovery tissue (Figure 3B). Overall, these results show that the altering of the recovery process of a PepGMV-infected plant by superinfection with PHYVV is associated with changes in viral chromatin epigenetic marks and a reduction of the levels of PepGMV genome methylation.

### 3.4. PepGMV Accumulation in Recovery Plants is Enhanced by PHYVV Encoded Rep and REn Proteins

PHYVV encodes six genes in two genomic components, A and B. Component A contains four genes: AC1 codes for a replication-associated protein (Rep), AC2 codes for a transcriptional activator protein (TrAP), AC3 codes for a replication enhancer protein (REn), and AV1 codes for the coat protein (CP). Component B contains two genes that are involved in viral movement, BC1 (MP, movement protein) and BV1 (NSP, nuclear shuttle protein) [40]. To test if a specific PHYVV gene could alter the host recovery stage of a PepGMV-infected plant, each gene was individually expressed in plants using a PVX-based expression vector. Expression of each PHYVV gene was verified by reverse transcription PCR using gene-specific primers (Figure S2).

**Figure 4:**
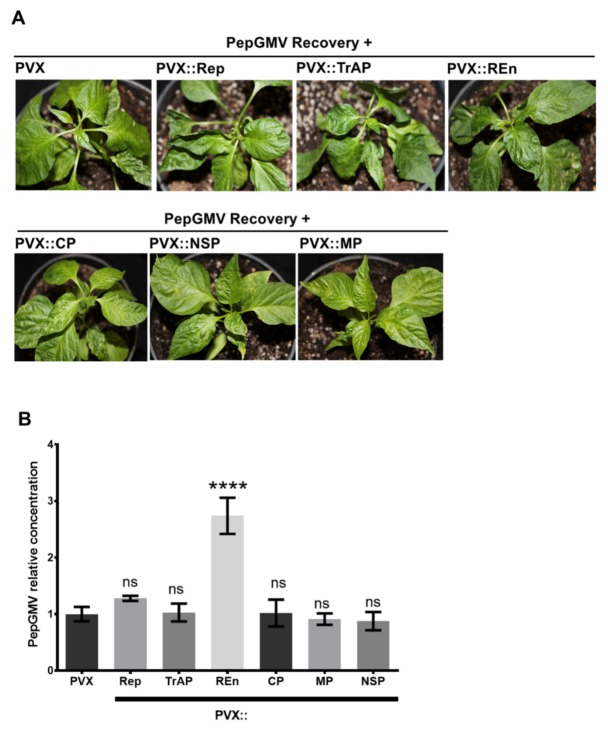
Effect of the expression of individual PHYVV genes in recovered PepGMV plants. (**A**) PepGMV recovered plants were inoculated with a PVX-vector expressing PHYVV individual genes. Ten days after inoculation, plant phenotype was analyzed and apical tissue collected for viral DNA quantification. (**B**) PepGMV DNA accumulation. PepGMV DNA levels were quantified by qPCR using the host gene CaEF1a for internal normalization. DNA concentration from different samples is expressed in reference to the value obtained with plant inoculated with PVX empty vector. Bars represent mean values of three independent biological replicates. Each replica is a pool of five plants. Error bars plotted to refer to the standard deviation of the mean. Asterisks indicate samples that are statistically different from the PVX empty vector sample (**** *p* < 0.0001; ns, not significant) determined by Dunnett’s multiple comparisons test.

As illustrated in Figure 4A, in contrast to the results obtained with the complete virus (PHYVV superinfection, Figure 1E), the expression of individual PHYVV genes is not enough to alter the recovery phenotype and induce the re-emergence of symptoms, i.e., to cause synergism. In addition to symptom evaluation, plants inoculated with PVX vector containing specific genes were also analyzed in terms of PepGMV DNA accumulation. Figure 4B shows the relative concentration of PepGMV DNA obtained in plants inoculated with the different PVX vectors using as reference the viral concentration obtained in plants inoculated with an empty PVX vector.

Plants inoculated with PVX-REn vector clearly show a higher accumulation of PepGMV DNA. Interestingly, plants inoculated with PVX-Rep showed a slight increase in viral titer that was not significant with the Dunnett’s multiple comparisons test, but using a pairwise comparison (empty PVX and PVX-Rep) and a Student’s *t*-test, the result was significantly different.

### 3.5. Expression of PHYVV REn Reduces the Levels of PepGMV DNA Methylation

**Figure 5:**
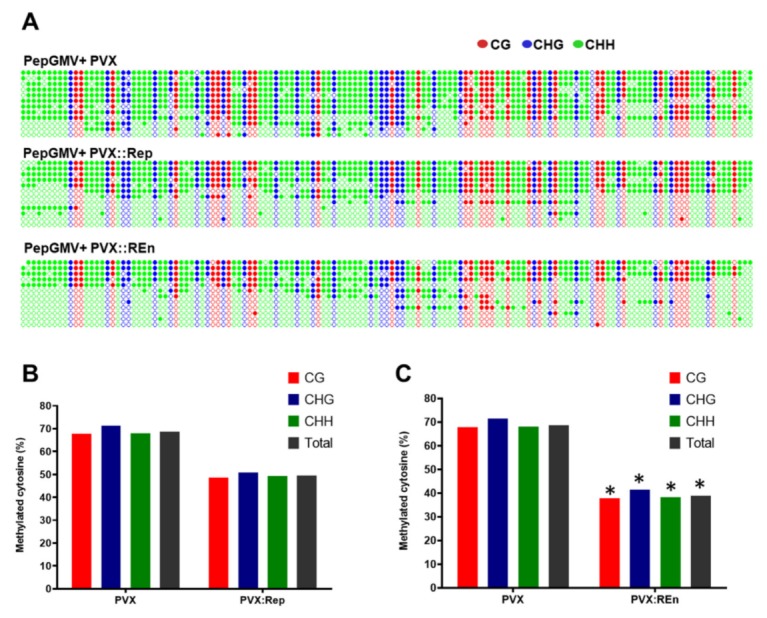
PepGMV DNA methylation level. PepGMV recovered plants were inoculated with PVX expressing PHYVV Rep or REn genes. Ten days after PVX inoculation apical leaves were collected for minichromosome extraction. DNA present in the minichromosomes was treated with bisulfite and used in a PCR assay to amplify and clone the viral fragment shown in Figure 2. Twelve clones per treatment were sequenced. (**A**) Cytosine methylation detected in individual clones. Dot graphics represent all cytosines present, 138 in total with CG (30) in red, CHG (28) in blue, and CHH (80) in green, filled circles indicate a methylated cytosine (mC). (**B**,**C**) Histograms show the percentage of mC residues in different sequence contexts. A Student’s *t*-test was performed using individual clones as data points. No significant difference was detected between control samples inoculated (PVX) and samples inoculated with PVX:Rep (**B**). On the other hand, a significant difference was observed between the control samples (PVX) and samples inoculated with PVX:REn (**C**) and it is indicated as * *p* < 0.05.

Since in PHYVV superinfected plants the PepGMV DNA is less methylated than present in recovered or PepGMV superinfected plants, we next asked whether the increase of PepGMV titters in plants that express PHYVV Rep and REn is associated with changes in the PepGMV DNA methylation status. To address that question, we performed bisulfite sequencing as described previously [14,18]. First, we determined that if PVX does not interfere with PepGMV DNA methylation, the PepGMV DNA methylation level remained high (68%) despite PVX presence. While in PepGMV recovered plants expressing PHYVV Rep, the methylation level decreased slightly in comparison to recovered (50% of cytosines are methylated) and in PepGMV recovered plants expressing PHYVV REn the PepGMV DNA methylation is similar to PHYVV superinfection (38%) (Figure 5B). These results show that both PHYVV Rep and REn proteins are involved in decreasing PepGMV DNA methylation suggesting a Rep and REn proteins role in defensive TGS pathway disruption.

### 3.6. PHYVV REn Is a TGS Silencing Suppressor

Transcriptional gene silencing acts as a defensive barrier against DNA viruses, in counter-defense, viruses encode suppressors of gene silencing to evade this mechanism [21].

*N. benthamiana* 16c-TGS line contains a GFP transgene downstream of a transcriptionally silenced CaMV 35S promoter. This line is frequently used to evaluate the TGS suppression activity [27,49]. To investigate if viral proteins Rep and REn, as well as the entire PHYVV, are able to suppress transcriptional gene silencing, we inoculated silenced *N. benthamiana* 16c-TGS plants with either PHYVV or recombinant PVX vectors expressing Rep or REn ORFs. Inoculated plants were evaluated under the UV light 10 dpi when plants inoculated with the virus had already developed symptoms.

**Figure 6:**
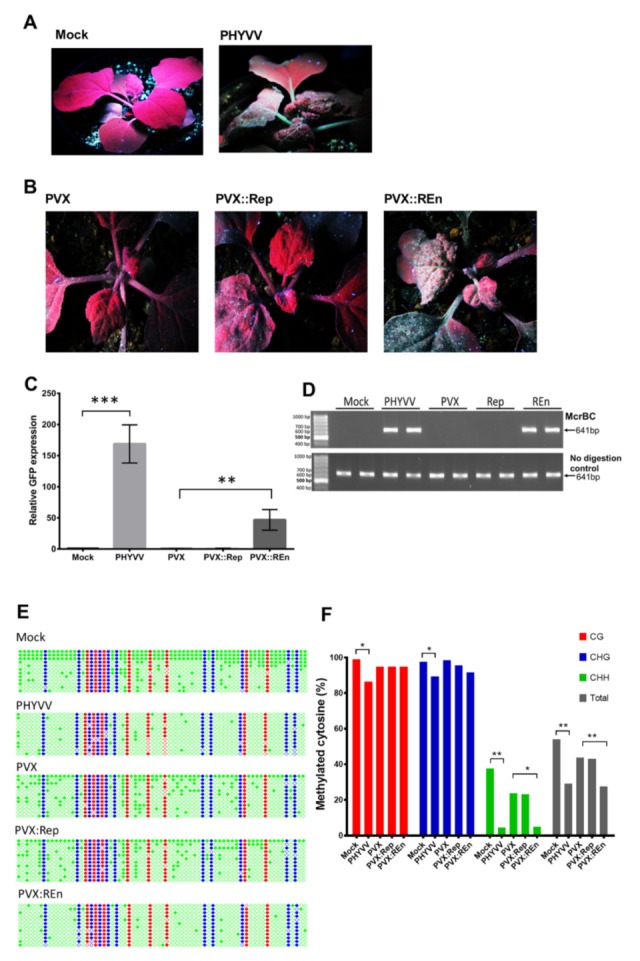
Transcriptional gene silencing (TGS) suppressor silencing analysis. (**A**) *Nicotiana benthamiana* 16c-TGS plants were inoculated by a biolistic procedure with PHYVV dimeric clones. Inoculated plants were evaluated at 10 dpi for green fluorescent protein (GFP) fluorescence using UV light. (**B**) *N. benthamiana* 16c-TGS plants were infiltrated with *Agrobacterium* strains harboring either an empty PVX-derived vector or a vector expressing PHYVV Rep (PVX::Rep) or REn (PVX::REn) proteins. Again, plants were evaluated for GFP fluorescence at 10 dpi using UV light. (**C**) Quantification of GFP expression by RT-qPCR. GFP expression levels were normalized to NbEF1a. Bars represent the mean values for three independent biological replicates, each replica contains a mix of three plants. Error bars represent standard deviation of the mean. Asterisks indicate pair samples that are statistically different (for PVX-PVX::REn: ** *p* < 0.01; for Mock-PHYVV: *** *p* < 0.001), determined by Student’s *t*-test. (**D**) Analysis of DNA methylation level of the 35S promoter by chop-PCR. Total DNA was extracted from vascular tissues, digested with methylation-dependent endonuclease McrBC, and used as template for the amplification of a 35S promoter segment using specific primers. Undigested DNA samples were used as control. (**E**,**F**) Analysis of DNA methylation level of the 35S promoter by bisulfite sequencing. Total DNA was extracted from vascular tissues and treated with bisulfite. PCR amplification and cloning of the amplicons was performed as explained in Section 2. Twelve clones per treatment were sequenced. (**E**) Cytosine methylation in individual clones. Dot graphics represent all cytosines (69) present in the fragment with the following contexts: CG (8) in red, CHG (11) in blue, and CHH (50) in green. Filled circles indicate a mC residue. (**F**) Histograms show the percentage of mC residues in all different sequence contexts. A Student’s *t*-test was performed using individual clones as data points. A significant difference between the samples in a pair is indicated as * *p* < 0.05 or ** *p* < 0.01.

The results indicate that PHYVV infection induces TGS suppression in 16c-TGS plants allowing the expression of GFP (Figure 6A). As shown in Figure 6B, the expression of REn (PVX::REn) is also able to suppress TGS. On the other hand, the expression of Rep, TrAP, or CP does not suppress TGS, and therefore, no GFP fluorescence was observed (Figure 6B and Figure S3A). In addition to the evaluation of fluorescence, GFP expression was also determined by RT-qPCR quantification of GFP transcripts (Figure 6C). To test the effect of the virus or viral protein on TGS suppression, we evaluated the levels of methylation of the 35S promoter that directs the expression of the GFP gene on the 16c-TGS plants. Bisulfite sequencing showed that the level of methylation of the 35S promoter in plants infected with either PHYVV or the vector PVX::REn was significantly lower than the level found in the same promoter from mock-inoculated plants. Similar results were obtained with a methylation-dependent (chop-PCR) assay (Figure 6D–F). On the other hand, although the expression of Rep resulted in a reduction of the levels of PepGMV DNA methylation on recovered pepper plants (Figure 5B), in this assay, the expression Rep was not able to either restore GFP expression (Figure 6C,D) or cause changes on 35S promoter methylation (Figure 6E,F).

### 3.7. PHYVV Rep Is a PTGS Silencing Suppressor

In plants, PTGS is another silencing mechanism that involves the degradation of mRNAs and is critical for host defense against RNA and DNA viruses. We tested whether Rep or REn proteins from PHYVV were able to suppress this silencing pathway. For this, we co-infiltrated plants of the line 16c of *N. benthamiana* with a mixture of either 35S:GFP plus 35S:Rep or 35S:GFP plus 35S:REn. This *N*. *benthamiana* line was selected to constitutively express GFP. Thus, the presence of a highly expressed GFP vector will induce silencing of the chromosomal GFP copy. The co-inoculation of a PTGS suppressor source will affect the silencing caused by the external, introduced GFP cassette [38].

**Figure 7:**
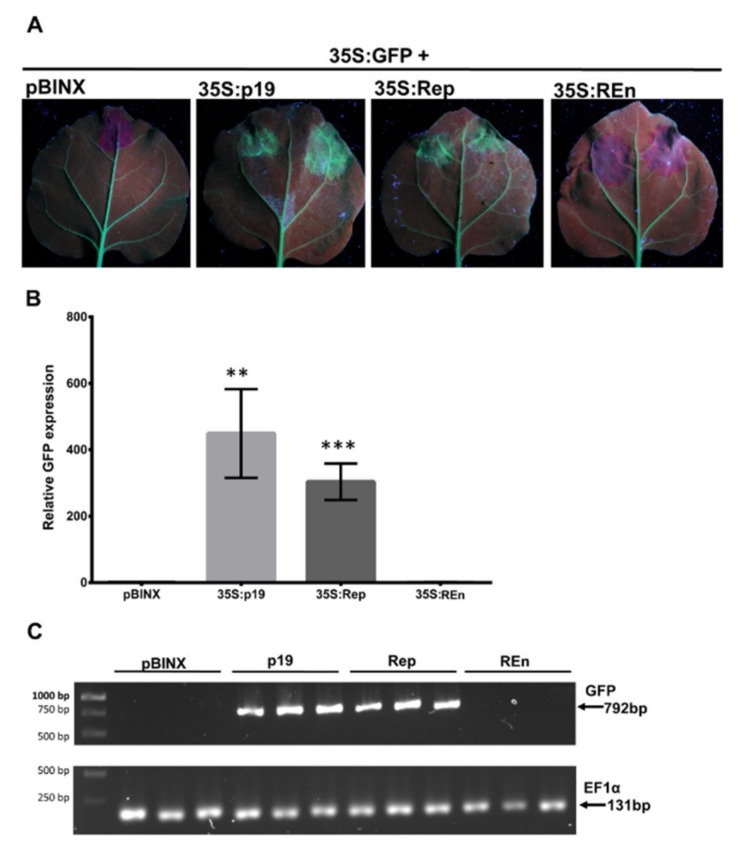
Post-transcriptional gene silencing (PTGS) suppressor silencing analysis. (**A**) *N. benthamiana* 16c plants were co-agroinoculated with a mixture of 35S:GFP plus pBINX empty vector or vectors expressing different viral proteins to be tested for PTGS suppression: 35S:p19, 35S:Rep, or 35S:REn. Five days after agroinoculation plants were evaluated for GFP fluorescence under UV light. (**B**) Quantification of GFP RNA expression by RT-qPCR. GFP RNA expression levels were normalized using the expression of the internal gene NbEF1a. Bars represent the mean values for three independent biological replicates. Error bars represent standard deviation of the mean. Asterisks indicate samples that are statistically different to pBINX (** *p* < 0.01; *** *p* < 0.001; ns, not significant), determined by Student´s *t*-test. (**C**) A RT-PCR assay was carried out to detect the expression of the GFP gene using primers that direct the amplification of the complete open reading frame (ORF). Total RNA was used for RT, followed by a PCR amplification (30 cycles). The amplification of host gene EF1α was used as internal control.

As expected, the agroinfiltration of 35S:GFP vector without a source of PTGS suppressor (empty pBINX) causes the silencing of GFP in the infiltrated area resulting in a lack of GFP fluorescence (Figure 7A). However, silencing is impaired by the co-infiltration of 35S:GFP with a vector expressing Rep protein (35S:Rep). In the infiltrated tissues, a good level of GFP fluorescence is observed even at similar levels to the one observed when 35SGFP is co-inoculated with a source of a well-characterized PTGS suppressor, p19, from a tombusvirus [52] (Figure 7A). This result indicates that PHYVV Rep protein is a suppressor of post-transcriptional silencing, PTGS. In parallel experiments with a vector expressing REn (35S:REn), no silencing suppression effect was observed (Figure 7A,B). Two additional PHYVV proteins, TrAP and CP, were also evaluated with this procedure. Similarly to REn, no evidence for a PTGS suppression effect was observed in those experiments (Figure S3B). In addition to the evaluation of GFP fluorescence, the PTGS suppression effect was also evaluated by quantification of GFP transcripts (Figure 7B,C). The levels of GFP transcripts detected correspond to the fluorescence observed in Figure 7A.

## 4. Discussion

Virus–host interactions are usually studied as a simple infection. However, mixed infections by two or more viruses are often found in nature [53]. Mixed infection between PepGMV and PHYVV has been studied in our laboratory from different points of view [7,8]. Often simultaneous inoculations (co-inoculations) are performed, leading to a synergistic effect without recovery, typically observed in a single infection [8]. We focused on the effect of superinfection by PYHVV in plants that underwent recovery following PepGMV infection. First, we inoculated plants with PepGMV, and once the plants had shown symptoms and the recovery process had been established, the plants were then superinfected with PHYVV (see Figure 1).

Previous works with PepGMV single infections have shown that the recovery process involves silencing of the viral minichromosome using both PTGS and TGS pathways (siRNA production, hypermethylation of the viral IR, minichromosome compaction, and association with histone repressive marks) [14,18]. In this study, we show that recovered, asymptomatic plants still maintain a reduced number of active, expressing PepGMV genomes that rapidly reinitiate a productive infection upon inoculation with the second virus, PHYVV resulting in a typical synergistic infection (Figure 1E,F). In synergistic conditions, the PepGMV population was characterized by methylation and histone modification levels similar to those of the symptomatic phase of a single PepGMV infection (Figure 2 and Figure 3).

Viral DNA methylation was demonstrated to be important for plant defense against geminiviruses. Hypermethylation of the viral genome has been associated with the recovery phenotype in different models of plant–geminivirus interaction [12,14,34,35,36]. In addition, mutants of RdDM are more susceptible to infection and show an exacerbated symptomatology compared to wild type plants [34].

The next question in this research was as follows: Is there a PHYVV protein responsible for synergism? In a previous study, a different approach to answer this question was used. In that study, each gene of the interacting viruses was independently mutated to study its role in the co-inoculation. It was determined that from the PepGMV side, TrAP was required for synergism, whereas on the PHYVV side, it was not possible to identify a gene solely required for synergism. Unfortunately, using that approach, Rep could not be evaluated since Rep mutations are usually lethal and no complementation occurs between PHYVV and PepGMV since iterons (Rep binding sites) are dramatically different [54]. In addition, it was complicated to evaluate REn since REn and TrAP ORFs are overlapped, thus, a mutation at the REn ORF also affects TrAP [8].

Given the need to express the viral proteins of PHYVV in pepper, the difficulty to obtain a pepper stable transforming line [55], and that *N. benthamiana* plants infected with PepGMV do not show recovery [7], we decided to express PHYVV proteins using an expression vector based on PVX.

Using PVX vectors, it was possible to show that TrAP, CP, MP, and NSP are not essential for synergism, whereas Rep and REn play important roles in the process by leading to an increase of PepGMV accumulation. For example, a 2.7-fold increase in viral DNA when expressing REn in comparison with the empty vector (Figure 4). It is interesting to mention, however, that even with that increase in the concentration of viral DNA, no symptom reappearance was observed. It is difficult to explain this result, but a possibility is that the number of infected cells by the PVX vector and the interacting PepGMV is different, therefore, the percentage of cells having both viruses simultaneously might be quite low. Another possible explanation is that even with the reported viral concentration, the viral titer needed to reach a putative threshold for symptoms is not reached.

Rep and REn are associated with viral replication. However, at least for Rep, its interaction with its own viral genome is quite specific. On the other hand, these viral proteins are reported as multifunctional, therefore, their participation in a synergism process might be through another pathway, with a lower virus specificity. We initially determined that an increase in PepGMV accumulation after superinfection with PHYVV of PepGMV recovered plants is associated with a reduction of viral DNA methylation (Figure 2). Next, we observed that PepGMV methylation level was lower in plants with a REn expression vector (38%) than in plants with a Rep expression vector (50%). Interestingly, the value obtained with REn is similar to the one obtained with PHYVV superinfection. These results suggested that these proteins could be suppressors of silencing.

Multiple geminiviral proteins have been associated with the gene silencing suppression, principally TrAP (AC2, C2, L2) [27,32,56,57,58]. In *African cassava mosaic virus* (ACMV), the suppressor of silencing is C4 like *Tomato leaf curl New Delhi virus* (ToLCNDV) [26,59], while in CLCuDV both TrAP and C4 have suppressor functions [24]. Another protein associated with suppression is V2 from *Mulberry mosaic dwarf-associated virus* (MMDAV), *Apple geminivirus* (AGV), *Tomato yellow leaf curl virus* (TYLCV), and *Beet curly top virus* (BCTV) [29,48,60,61,62]. Therefore, despite belonging to the same viral family each virus has different suppressor silencing proteins, so this function cannot be generalized in all geminiviruses and it is necessary to analyze each individual virus.

Although PHYVV was described almost 30 years ago [40,63], this is the first analysis in search of silencing suppressors encoded in this virus. To determine silencing suppressor activity, we used the *N. benthamiana* lines 16c and 16c-TGS, which have been widely used for this purpose [27]. As shown in Figure 6 and Figure 7, PHYVV Rep is not able to suppress TGS but might be able to for PTGS. In contrast, REn is shown to suppresses TGS, but not PTGS in *N. benthamiana.* These viral proteins act as a counter defense at different levels. Further research should be done to investigate in detail how they suppress gene silencing.

The ability of geminiviral Rep to suppress silencing has been poorly studied. TYLVC Rep decreases the expression of Met1 and CMT3 DNA methyltransferases, in addition to decreasing methylation at host DNA *loci*, such as FWA or a reporter TGS-silenced gene (GUS gene), resulting in the release of TGS arrest and the expression of the corresponding genetic element [30]. Regarding PTGS, we obtained some evidence that PHYVV Rep might have suppressive activity. There are no reports of a Rep protein from another begomovirus showing PTGS suppression. However, a Rep protein from the mastrevirus *Wheat dwarf virus* (WDV), was reported with PTGS suppression activity [25].

Research on the role of REn protein is scarce. REn has been reported as an enhancer of replication since REn mutants show a decrease in viral replication. Interestingly, viral replication can be restored by complementation with REn from other viruses suggesting a low specificity [64,65]. This enhancement in viral replication is associated with the interaction between REn, Rep, and PCNA [65,66]. Rep and REn have been shown to interact with Replication Protein A (RPA) [67,68], a single-stranded DNA binding protein involved in several processes, including maintenance of TGS of transposon elements [69,70]. RPA silencing resulted in enhanced TYLCSV infection [71]. Regarding the other activity, REn from AbMV was suggested to avoid early TrAP brake of viral replication [72].

Contradictory results on its interaction with proteins that affect chromatin have been reported with *Tomato leaf curl Kerala virus* [68] and TYLCV [73]. Nevertheless, geminiviral REn has never been reported as a TGS suppressor. The evidence shown here with PHYVV REn and this activity warrant further research to facilitate our understanding of these multifunctional proteins, and special emphasis is required to study the interaction between viral proteins, i.e., Rep-REn.

In summary, in a recovered plant the silencing mechanisms are activated to restrict viral replication through DNA methylation. However, when plants are superinfected with PHYVV these mechanisms are not sufficient to maintain the PepGMV genome repressed, and therefore synergism is triggered. The combined presence of Rep, Ren, and TrAP from two geminiviruses might generate a strong silencing suppressive response that the plant is not able to counteract. Nevertheless, these interactions might also help us to understand why these viruses keep sharing the same geographical distribution after many years without any indication that one might gradually eliminate the other one as it has been reported in many areas with the arrival of a “foreign” entity.

Finally, it could be interesting to identify the host (pepper) proteins interacting with PHYVV Rep and REn proteins by using heterologous systems as the Yeast Two-hybrid system (Y2H) or Mass spectrometry-based systems in order to elucidate this novel mechanism of silencing suppression.

## Supplementary Materials

The following are available online at https://www.mdpi.com/1999-4915/12/3/286/s1, Table S1: Primers, Figure S1: Schematic representation of PHYYV genome, Figure S2: Expression of PHYVV individual genes, Figure S3: Suppressors silencing analysis.
