# Supplementary material for: Superinfection by PHYVV Alters the Recovery Process in PepGMV-Infected Pepper Plants
Source: Viruses. 2020 Mar 5;12(3):286. doi: 10.3390/v12030286 (PMC7150747; doi:10.3390/v12030286)
Supplement: Supplementary file 1 [file viruses-12-00286-s001.pdf]

Table S1. Primers

Name	Sequence	Purpose
qPG F	ccactcacaatagagggctgag	To quantify PepGMV
qPG R	tgacaggcgggatgtgattg	To quantify PepGMV
Ca EF1 F	ctggtcgagagcctcaag	To quantify C. annuum EF1
Ca EF1 R	ctcaagaaggtcggttacaac	To quantify C. annuum EF1
VC F	taaatatattaaaaaaatatttttac	Bisulfite sequencing PepGMV
VC R	tttaggtatatttgggtttttata	Bisulfite sequencing PepGMV
Rep PH F	cctctcttattacaaatatgccattac	To amplify PHYVV Rep ORF
Rep PH R	gtctctttgcaaatctatggcg	To amplify PHYVV Rep ORF
Trap PH F	aaaaataggagcctcaatgactg	To amplify PHYVV TrAP ORF
Trap PH R	catgacaagcaatttaaatctatttaag	To amplify PHYVV TrAP ORF
Ren PH F	gtgttaacaatggatttacgcac	To amplify PHYVV Ren ORF
Ren PH R	cgggtctatttttatgactcgataatg	To amplify PHYVV Ren ORF
CP PH F	cctttaattcaaaatgcctaagcg	To amplify PHYVV CP ORF
CP PH R	acaaactttattaattcattatcgagtc	To amplify PHYVV CP ORF
MP PH F	gccaaattttcatatatggattcatg	To amplify PHYVV MP ORF
MP PH R	ttattatcttagcgacttcggttg	To amplify PHYVV MP ORF
NSP PH F	aatatgtattctactagatttagacgtg	To amplify PHYVV NSP ORF
NSP PH R	aaactacgcaagtcaattatttct	To amplify PHYVV NSP ORF
qRepPH Fw	cgtctcgctcaactacaaaacc	To verify Rep expression
qRepPH R	atcggttgttcgtgcattgg	To verify Rep expression
qTrAP F	gcaaagagacagatacgacgtagaagg	To verify TrAP expression
qTrAP R	gtacattggcgtctccactagc	To verify TrAP expression
qRenPH Fw	tacccatcactgcagctcaag	To verify Ren expression
qRenPH R	cctcatgttgtggtttgctctg	To verify Ren expression
qCpPH Fw	cctcagcttgggttaatcgc	To verify CP expression
qCpPH R	ccttacagggaccttcacaacc	To verify CP expression
qMPPH Fw	tcagcatccatgtcaaggttc	To verify MP expression
qMPPH R	atgcgctatgtccttggttg	To verify MP expression
qNSPPH Fw	ggtgatgctaaacgacgtcag	To verify NSP expression
qNSPPH R	tgggctgagtcttacacagttg	To verify NSP expression
qGFP F	gagggatacgtgcaggagag	To quantify GFP
qGFP R	gatcctgttgacgagggtgt	To quantify GFP
Nb EF1 F	gattggtggtattggaactgtc	To quantify N. benthamiana EF1
Nb EF1 R	agcttcgtggtgcatctc	To quantify N. benthamiana EF1
35S Chop F	gattcaggactaactgcatcaag	To amplify 35S promoter
35S Chop R	ttgcgaaggatagtgggattgtg	To amplify 35S promoter
CaMV 35S-Bis-F	aaggyaagtaatagagattggagt	Bisulfite sequencing PepGMV
CaMV 35S-Bis-R	ccttccttttccactatcttcacaat	Bisulfite sequencing PepGMV
GFP F	atgaagactaatctttttctctttc	To amplify entire GFP ORF
GFP R	ggatcctttgtatagttcatccatgc	To amplify entire GFP ORF
PVX F	tggcttgcaaactagatgcaga	To amplify cloning site in pGR107
PVX R	accctatgggctgtgttgt	To amplify cloning site in pGR107

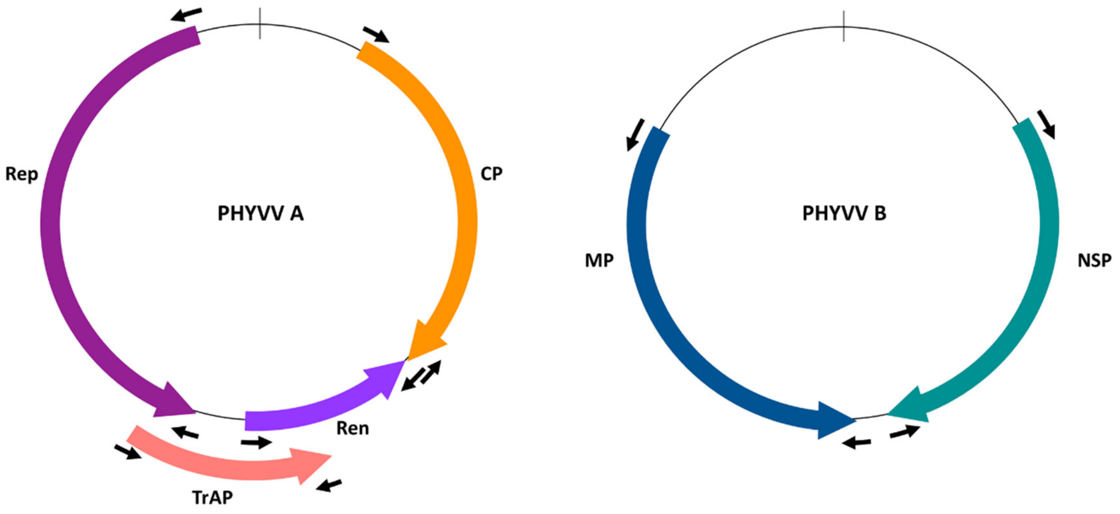

Figure S1. Schematic representation of PHYYV genome. PHYVV encodes six genes distributed into two molecules called components A and B. The A component contains the capsid protein gene CP in the virion sense strand, whereas the complementary sense strand encodes Rep, TrAP, REn. The B component encodes two movement proteins, NSP in the virion sense strand and MP the complementary sense strand. Primers used to amplify each gene are schematized by black arrows.

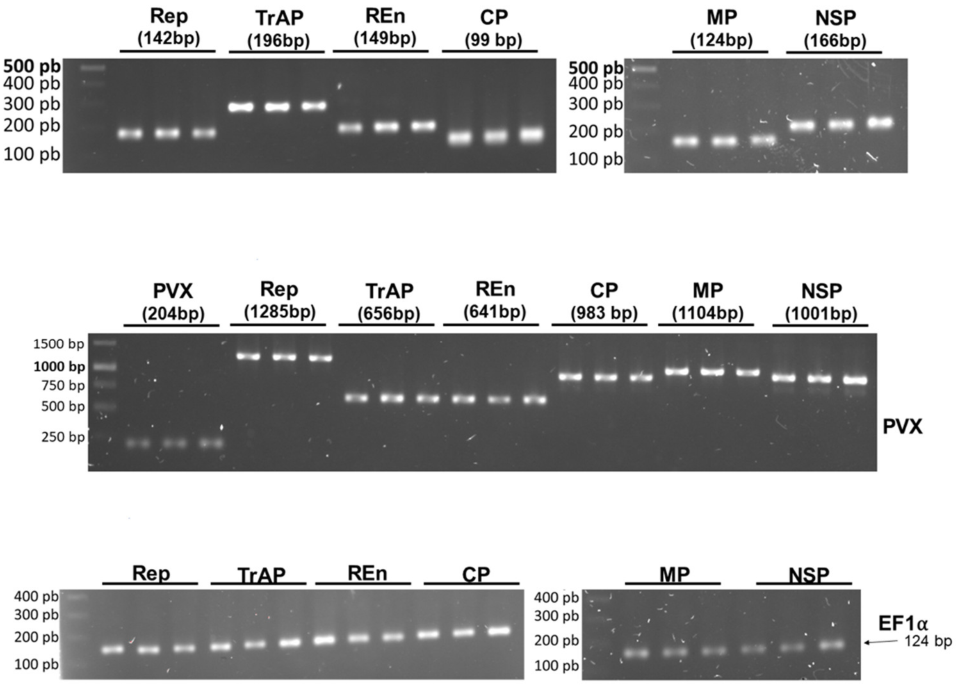

Figure S2. Expression of PHYVV individual genes. RT-PCR was carried out to detect PHYVV gene derived transcripts. Total RNA was used for RT, followed by PCR amplification (25 cycles). The results shown in all panels represent three independent plants in each case.

Upper panel primers: Rep (qRepPH Fw, qRepPH R), TrAP (qTrAP F, qTrAP R), REn (qREnPH Fw, qREnPH R), CP (qCPPH Fw, qCPPH R), MP (qMPPH Fw, qMPPH R), NSP (qNSPPH Fw, qNSPPH R). The expected sizes of the amplicons are also shown in the panel: Rep 142pb, TrAP 196bp, REn 149bp, CP 99bp, MP 124 bp, NSP 166bp).

Middle Panel Primers: Primers to direct the amplification of the cloning site segment of PVX vector (PVX F, PVX R). This panel shows the expected size, in bp, of RT-PCR amplification product from: PVX vector without insert (204), or PVX vector with inserts from different PHYVV genes (Rep 1285, TrAP 656, REn 641, CP 983, MP 1104, NSP, 1001).

Lower panel primers: Primers used to direct the amplification of EF1α gene used as internal control in qPCR (NbEF1 F, Nb EF1 R). Expected size of amplified product 124 bp.

All primers sequences are included in Figure S1.

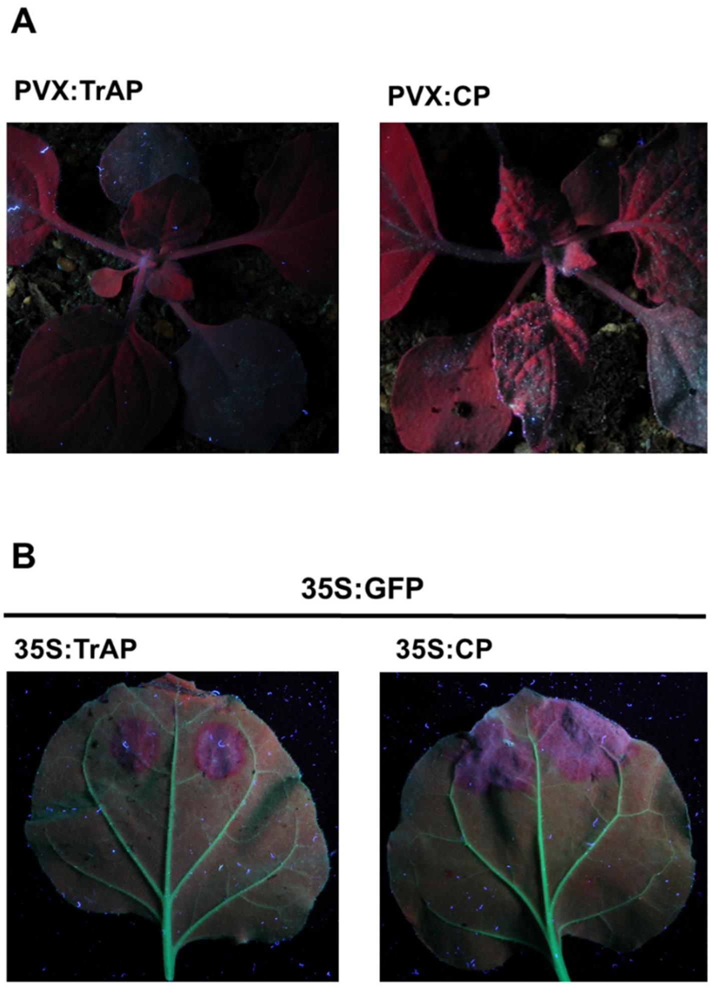

Figure S3. Suppressors silencing analysis. (**A**) TGS suppressor silencing analysis. *N. benthamiana* 16c TGS plants were infiltrated with *Agrobacterium* strains harboring PVX empty vector or expressing PHYVV TrAP, or CP proteins, photographs were taken under UV light at 10 dpi. (**B**)PTGS suppressor silencing analysis. *N. benthamiana* 16c plants were co-agroinoculated with a mix of 35S:GFP plus with a mix of 35S:GFP plus, 35S:TrAP or 35S:CP. Photographs were taken under UV light at 5 dpi.
